# Nano-pesticides: the lunch-box principle—deadly goodies (semio-chemical functionalised nanoparticles that deliver pesticide only to target species)

**DOI:** 10.1186/s12951-021-01216-5

**Published:** 2022-01-04

**Authors:** J. J. Scott-Fordsmand, L. F. Fraceto, M. J. B. Amorim

**Affiliations:** 1 Department of Bioscience, Aarhus University, 8600 Silkeborg, Denmark; 2 Department of Environmental Engineering, São Paulo State University, Sorocaba, 18087-180 Brazil; 3 Department of Biology & CESAM, University of Aveiro, 3810-193 Aveiro, Portugal

## Abstract

Nature contains many examples of “fake promises” to attract “prey”, e.g., predatory spiders that emit the same sex-attractant-signals as moths to catch them at close range and male spiders that make empty silk-wrapped gifts in order to mate with a female. Nano-pesticides should ideally mimic nature by luring a target and killing it without harming other organisms/species. Here, we present such an approach, called the lunch-box or deadly-goodies approach. The lunch-box consists of three main elements (1) the lure (semio-chemicals anchored on the box), (2) the box (palatable nano-carrier), and (3) the kill (advanced targeted pesticide). To implement this approach, one needs to draw on the vast amount of chemical ecological knowledge available, combine this with recent nanomaterial techniques, and use novel advanced pesticides. Precision nano-pesticides can increase crop protection and food production whilst lowering environmental impacts.

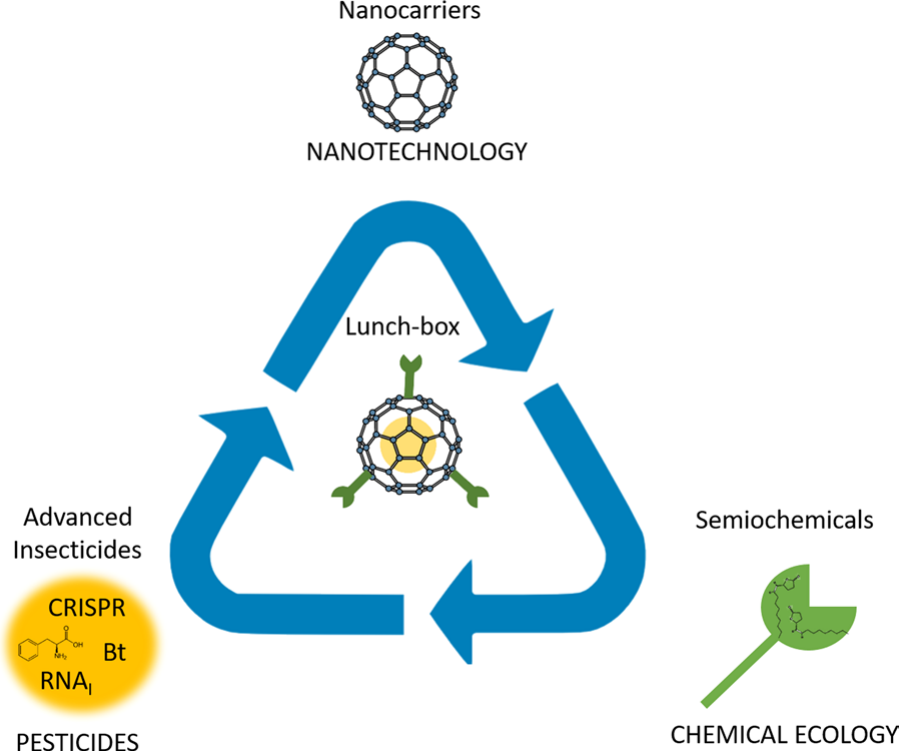

## Background

Some of the biggest challenges for modern society, e.g., sustainable increase crop protection, elimination of vector borne diseases, all whilst keeping or promoting biodiversity. For example, FAO estimate that 20–40% of all crop production is lost to pests [1] and WHO estimate that globally vector borne diseases are responsible for 17% of all infections [2]. To reduce the damage caused by these pests conventional pesticides are widely used. These pesticides are spread directly in nature as chemicals in various formulations, in the order of three billion tonnes [3, 4]. This application approach entails a uniform cover of chemicals on the target environment at a defined time. However, a large proportion of the pesticide never reaches the target organisms but instead reach non-target organisms, ground water, etc. To deal with some of these issues, progress has been made in the area of nano-pesticides, aimed at reducing general spreading of the pesticide and providing timed release, hence reducing overall emissions [5]. In recent years, nano-delivery-systems, e.g., chitosan, pectin or zein-nano-carriers containing pesticides, have been developed as a way to use smaller amounts of pesticides. The aim is to distribute the pesticide in a more time resolved and targeted way [6–12]. In this approach, the encapsulated pesticide is released from its nanocarrier upon an environmental trigger, e.g., moisture. The pest organisms (or any other animal) may randomly contact the released pesticide or consume the encapsulated material. However, no study has yet examined whether it is possible to entice a specific pest-species to contact the nanocarrier.

We describe a concept, the nano lunch-box approach that eliminates the described randomness of encountering the pesticide by combining a nano-delivery system with a semio-chemical, i.e., pheromone, allomone, kairomone or synomone. The aim is to make the pest organism wish to approach the encapsulated pesticide, i.e., using the attract-to-kill approach [13, 14] at the nanoscale. The attract-to-kill approach has been commonly used bait traps [13], but never on nanocarriers. The pest organism sets out on a deliberate quest to find the lunch-box, without knowing the contents are deadly. Hence, the target is to make an attractive lunch-box that contains a targeted killer. The attractiveness is obtained by anchoring species-specific semio-chemicals on the surface of the nanocarrier. The box is a nanocarrier of a highly palatable material that can be digested in the midgut of the target pest. The killer is a pesticide that is species and life-stage specific.

From a purely natural perspective, the lunch-box approach is not very novel since nature has many examples carriers with chemotaxis—the novel part here is that human many be able to utilise this approach. In line with this, Nature contains many examples where “fake promises” are used to attract “prey”, e.g., predatory spiders that emit the same sex-attractant-signals as moths for catching them at close range [15], male spiders that make empty silk-wrapped gifts in order to mate with a female [16], and plants that emit an odour that attracts certain species of insects [17]. Hence, the lunch-box approach mimics nature and we can draw on a vast amount of chemical ecological knowledge in the development of this approach.

**Fig. 1:**
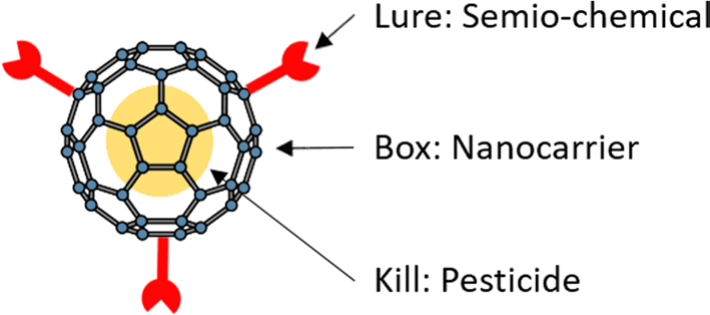
Principle of components of the Lunch-Box approach. Three components are required (1) the lure (chemical ecology) based on semio-chemicals, (2) the box (nanotechnology), which include novel nanocarriers made of palatable natural materials, and (3) the kill (pesticides), which can include smart pesticide that are more targeted and ensure a protected environment until it reaches the target species. *For visual reasons we use a bucky-ball to illustrate the nano-container, we will not use bucky-balls but polymers but in a drawing, these would simply be opaque

The lunch-box approach consists of three main steps—(1) the lure, (2) the box and (3) the kill (Fig. 1).

## The lure

**Fig. 2:**
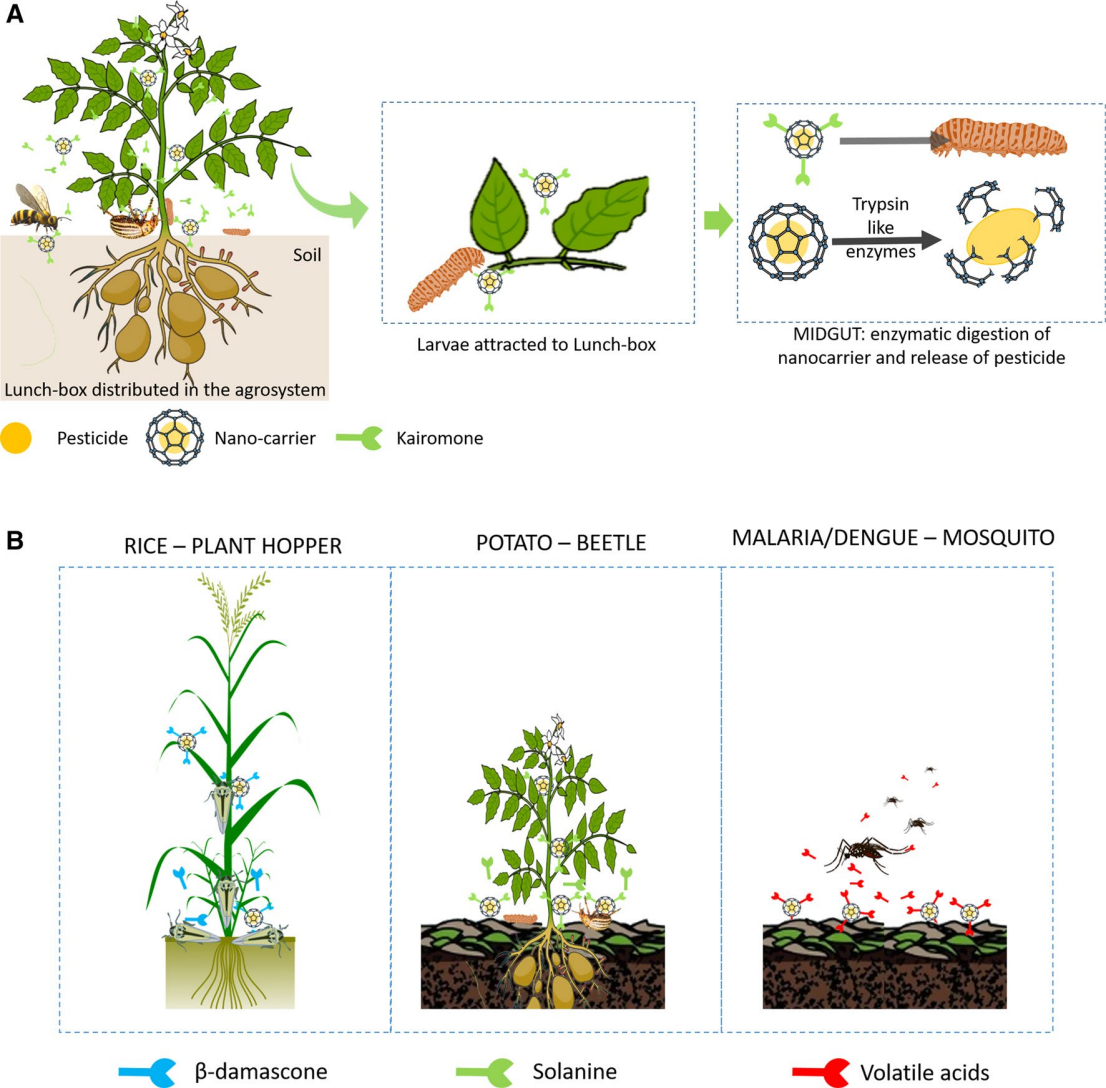
The nano-pesticide lunch-box principle. **A** Example with the Colorado potato beetle (*Leptinotarsa decemlineata*). The potato beetle almost exclusively targets night-shade plants (*Solanum*, containing the poisonous Solanine). A lunch-box covered with attractive chemical cues similar to the plant (kairomones) can be used to attract the beetle [77, 78] or pheromones that promote beetle aggregation [79]. While the beetle (and the larvae) is attracted, other organism (e.g., bees) will be repelled or not attracted. The kill could be Bt crystalline proteins or RNAi [80]. **B** Using various species. A similar approach can be used for various insect, e.g., planthoppers, beetles, and mosquitoes in each case the nanocarrier has different semio-chemicals attached to surface [29]. *For visual reasons we use a bucky-ball to illustrate the nano-container, we will not use bucky-balls but polymers but in a drawing, these would simply be opaque

The lunch-box approach requires that the pest organism senses an advantage in finding or being close to the “lunch” and is therefore lured into this “belief”. There are no previous reports on this approach, although studies have shown that pheromones can be embedded in polymer fibres [18] or nanogels [19]. The approach involves anchoring highly attractive chemicals on the surface of a nanocarrier. Potential attractants include semio-chemicals, e.g., volatile compounds that signal attraction and mating, that signal food, or more general host detecting chemicals. These chemicals can be highly species specific, can be detected by organisms even at very low concentrations and can induce a response that overrides many of the natural “fears” within an organism [14, 20]. Semio-chemical compounds are known for some of the main pest species [21] (e.g., see lists of the European Food Safety Authority (EFSA) published October 2019 of top pests for plant species [22], or for well-known global human pests [23–31]). For species where these compounds are not yet known, novel sensitive detection techniques [32] or reverse chemical ecology [27] can be used to identify, effective compounds. Once attractants (semio-chemicals) have been identified, novel synthetic biology methods (e.g., engineered yeast cultures) can be used to produce sufficient amounts at low cost [33, 34]. Obtaining an optimal anchoring (from a loading- and release-rate perspective) is important. However, binding semio-chemical cues to the surface of a nanocarrier may be challenging, e.g. when trying to maintain the correct chirality and general stereochemistry of the attached chemicals [15, 35]. Gonçalves et al. [36] showed that it was possible (via anchors) to bind odours to functionalised cotton surfaces in clothes. When the clothes were worn, the odour was released due to pH changes induced by sweat. The anchors were in this case carbohydrate-binding modules with an attach spacer (repetition of glycine-glutamine residues) to confer conformational mobility [36]. Such a pH dependent approach can also be used for pheromones, which show pH dependent reversed binding to receptors via the C-terminal [37]. It may, depending on the specific cases, be considered whether release is necessary (and how much) or whether is it enough for the semio-chemical to be attached to the “box”. For example, if the media (air, soil or water) transport the nano-pesticide to the pest species, the pest species will detect the semio-chemical loaded carrier and a release of semio-chemicals may not be advantageous. Previous studies in related areas are: (1) studies on the surface modelling and functionalisation of nanomaterials providing information on how strong and weak binding sites can be formed on the nano surfaces [38–41], e.g., via cross-linker [42]. Further, models show that nanocarriers, depending on the size and material, may contains tens of thousands surface atoms, i.e. potential functionalisation sites [43]. (2) Studies on nanomaterial (bio-)corona interactions providing information on how organic molecules bind to nano-surfaces [44, 45]. (3) Studies on the reversible binding of pheromones to insect surfaces revealing how semio-chemicals can be reversibly bound to nanocarriers [21]. By integrating these three areas, we showed that it was possible to load semio-chemicals on nanocarriers and at the same time ensure their controlled release (Fig. 2). The reader may be reminded that the above “binding semio-chemical cues to the surface of a nanocarrier may be challenging” refers to challenging for humans, but for nature this is ubiquitous occurring. We may obviously also learn from nature here, e.g., volatile compounds or semio-chemicals from surfaces of bacteria or pollen [46, 47].

## The box

In this approach, if a pest organism is attracted to the lunch-box, it must then “open” it, which requires that the carrier is made of a palatable/digestible material, e.g., cellulose or pectin. The “opening” could be triggered by gut digestive enzymes or physical–chemical parameters [48–50]. Hence, the material properties are important for the carrier’s stability, the potential to be opened, and, in particular, the timing of the box’s opening is crucial. The encapsulation should consider materials already present in nature, i.e., nanomaterials based on compounds, such as sugars or polysaccharides (cellulose derivatives, chitosan, pectin, lignin, etc.), proteins (zein, casein, etc.) or inorganic materials (silica, etc.). These may be produced/extracted either directly or by recombinant methods [51, 52]. Many natural materials have properties suitable for nanocarrier systems and are degradable by enzymes present in organisms. Hence, they are good candidates for promoting the release of active ingredients in site-specific pest-control. In a recent review, Fraceto and co-workers [53] presented an overview of the development of stimuli-responsive nanomaterials that can be used for nanocarriers. Such systems can enable the site-specific release of active ingredients (insecticides, repellents, acaricides, etc.) under biotic (fungi, insects, weeds, nematodes, etc.) and abiotic stress conditions (pH, temperature, drought, salinity, etc.). Most of these site-specific release systems were inspired from drug delivery and food science research, whereas systems that promote agricultural release applications are still at an early stage. A few papers have reported that enzymes present in the salivary glands and midgut of larvae and insects are good candidates for triggering the release owing to the presence of carbohydrates, glycans and proteases [54–58]. For example, Oliveira et al. [58] showed that carrier systems based on zein nanoparticles (loaded with botanical insecticides) had a potential dual advantage: (1) when consumed by larvae, they released the active compound (trypsin based hydrolyse in the midgut), and (2) when not consumed, there was only very slow release of the active compound. In another example, Kaziem et al. [59] developed a system based on cyclodextrin anchored in hollow mesoporous silica loaded with avermectin where the release was controlled by the α-amylase activity of *Plutella xylostella*. In summary, strategies to deliver pesticides using site-specific nanoparticles are extremely interesting because they enable targeted effects on an organism whilst avoiding non-effective release of the active compound.

## The kill

Once the lunch-box is open, the pesticide can perform its action at the target site without harming other organisms. The approach goes beyond conventional chemicals and allows the use of more benign and sophisticated approaches. For example, Bt (*Bacillus* *thuringiensis*) can be used against various pest species, Bt by inducing lethal midgut lesions, which kills the organism. Hence, Bt can be encapsulated in a pheromone-loaded carrier and used as an insecticide [60], a nanocarrier if the crystal is used and a microcarrier if the spores are used. Within the nanocarriers, novel natural or biosynthetic “compounds” can also be employed, e.g., natural chemicals [7], small-molecule agonists [61], or novel synthetic RNAi virus like strings [62–64]. Alternatively, they can be used as a platform for delivering CRISPR ribonucleoprotein for gene editing in the target [65] as for example used for vector-borne diseases from Mosquitoes [29, 66]. With this system, the pesticides can be more accurately targeted and are generally less damaging than conventional pesticide chemicals because the carrier system can protect and ensure proper functioning. Since nanocarriers may cross the midgut membrane, the lunch-box may even be designed to target specific tissues before release, although development of the latter may take longer. Obviously, by controlling the size of the carrier, e.g., between nano and micro size, it is possible adjust what can be inside the carrier but also to enhance or inhibit cellular internalisation [67]. Finally, the expiry date of the kill substance should be considered, i.e., the degradation rate of the kill material should be faster than that of the nanocarrier (when not triggered) as this will also help to prevent undesirable release of unused pesticide [68].

## Lunch-box example—based on combining previous research

Rice is one of the world’s most important foods, with 750 million tonnes being produced globally, but rice is infested by numerous pests [69, 70]. We here show how the lunch-box principle can be applied to rice by using essential oil semio-chemicals, polymer nanocarriers and various pesticides.

### The lure

Kuhnt et al. [71] showed a sustained rose fragrance (semio-chemical) release from functionalised cellulose nanocrystals (CNC) (10–30 nm × 100–300 nm) decorated with β-damascone. They linked the fragrance via a short thioether that served to bind the fragrance molecules to the hydroxyl bonds on the CNCs. The release was pH dependent, controlling release under neutral or basic conditions. The chemical group which damascene belong to, i.e., damascenone, contains closely related chemical compounds, i.e., damascene and ionone, hence this indicates that β-ionone may also be bound to the cellulose by the same thioether technique. The β-ionone is an attractant [72] for the white-backed planthopper, *Sogatella furcifera* (Horváth) (Hemiptera: Delphacidae), one of the main agricultural insect rice pests in China.

### The box

The CNC is made of cellulose, which is a natural polysaccharide with many hydroxy groups on the surface. To this group belong other polysaccharides such as chitosan, alginate and pectin, which also contain many hydroxyl groups on the surface. These polysaccharides are well known as nanocarriers for pesticides [73].

### The kill

The polysaccharide nanocarriers have been loaded with a wide variety targeted insecticide, e.g., the neonicotinoids thiamethoxam (nanocellulose carrier) [74], the bactericide Iprofloxacin-HCl (nanochitosan carrier) [75], the botanical compound Geranolium (nanochitosan carrier) [76], and in human health studies the gene silencing siRNA [64]. These polysaccharide nanocarriers also show controllable release properties [73].

Hence, we here outline a lunch-box pesticide, where an attractant in the form of essential oils are attached to polysaccharides nanocarriers, a nanocarrier that is able to deliver a wide range of traditional and more benign pesticides. This lunch-box pesticide can target a pest species (white-backed planthopper, *Sogatella furcifera)* affecting one of the main global agricultural crops (rice, *Oryza glaberrima*/*sativa*), which has shown resistance to traditional pesticides. The lunch-box system will enable delivery of more targeted and at the same time more generally benign pesticides directly to the pest species.

## Nano-pesticide

The global pesticide usage has been estimated to two billion tonnes, but predictions are that this usage has currently increased to 3.5 billion tonnes [3, 4]. The current non-specific pesticide approach is to spread chemicals over a vast area, reaching both target and non-target organisms [81–85].

**Fig. 3:**
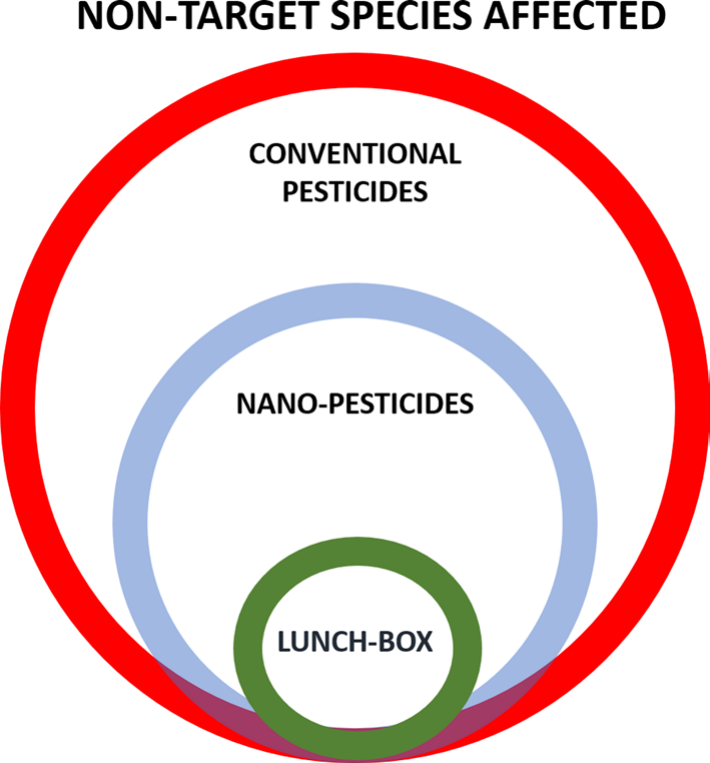
The relative amount of non-target species affected by the various pesticide approaches. The conventional chemical approach (yellow) where many non-target species are affected, to pure nanocarriers (grey) that can limit collateral damage by being external trigger dependent, to the lunch-box approach (green) which will be even more specific because it is designed to attract only the target species

Thus, a large proportion of the pesticide does not reach the pests efficiently and may promote resistant populations. Novel methods of using naked or functionalised nanocarriers containing pesticides reduce this problem and enable novel pesticides to be used. However, these novel methods are not species specific and rely on random encounters. The lunch-box or deadly goodies concept eliminates this randomness by using species-specific attractants on the nanocarriers, which may also be life-stage specific (Fig. 2A) and can target different species (Fig. 2B). Hence, the lunch-box approach can (1) be highly species-specific, (2) lower pesticide use, (3) utilise “benign” pesticides, (4) ensure the diversity of other species, and (5) decrease pesticide residues in food and the environment (Fig. 3). Nevertheless, it is important also for such an approach to fully understand the life-cycle fate of the nano-carriers/-nanopesticides. How do they impact (benefits/risk) the environment in which they are introduced, are they indeed able to provide better sustainability and less collateral ecotoxicity.

## Wider perspective

The lunch-box approach can also help to sustain beneficial species (e.g., important pollinators). For example, bees are reported to be increasingly affected by parasites (e.g., host specific *Crithidia biomb*, *Paenibacillus larvae*, *Nosema ceranae*, etc*.* [86]). Hence, a bee specific lunch-box that contains anti-parasitical compounds could help the bee population and in turn pollination while minimising effects on other species. The above approach is, to some extent, in line with the principle of nano-medicine, utilising functionalisation to reach the target, e.g., functionalised zein or virus-like nanoparticles [8, 87–89]. However, for nano-pesticides, we aim to make the pest organism do the work of coming to the “medicine/cure”.

In summary, compared to present approaches the lunch-box concept seems to be highly promising for developing precision nano-pesticides that enable targeted release, increased efficacy and avoid widespread undesirable effects of pesticides. The approach benefits from the interplay between chemical, nano-technological, and ecological sciences.

## Data Availability

All data are included.

## References

[1] New standards to curb the global spread of plant pests and diseases. http://www.fao.org/news/story/en/item/1187738/icode/. Accessed 1 Aug 2021.

[2] Vector-borne diseases. https://www.who.int/news-room/fact-sheets/detail/vector-borne-diseases. Accessed 1 Aug 2021.

[3] Zhang W (2018). Global pesticide use: profile, trend, cost/benefit and more. Proc Int Acad Ecol Environ Sci.

[4] Sharma A, Kumar V, Shahzad B, Tanveer M, Sidhu GPS, Handa N, Kohli SK, Yadav P, Bali AS, Parihar RD (2019). Worldwide pesticide usage and its impacts on ecosystem. SN Appl Sci.

[5] Lowry GV, Avellan A, Gilbertson LM (2019). Opportunities and challenges for nanotechnology in the agri-tech revolution. Nat Nanotechnol.

[6] Grillo R, Clemente Z, de Oliveira JL, Campos EV, Chalupe VC, Jonsson CM, de Lima R, Sanches G, Nishisaka CS, Rosa AH (2015). Chitosan nanoparticles loaded the herbicide paraquat: the influence of the aquatic humic substances on the colloidal stability and toxicity. J Hazard Mater.

[7] de Oliveira JL, Campos EVR, Germano-Costa T, Lima R, Vechia JFD, Soares ST, de Andrade DJ, Goncalves KC, do Nascimento J, Polanczyk RA, Fraceto LF (2019). Association of zein nanoparticles with botanical compounds for effective pest control systems. Pest Manag Sci.

[8] Patra JK, Das G, Fraceto LF, Campos EVR, Rodriguez-Torres MDP, Acosta-Torres LS, Diaz-Torres LA, Grillo R, Swamy MK, Sharma S (2018). Nano based drug delivery systems: recent developments and future prospects. J Nanobiotechnol.

[9] Sun Y, Liang J, Tang L, Li H, Zhu Y, Jiang D, Song B, Chen M, Zeng G (2019). Nano-pesticides: a great challenge for biodiversity?. Nano Today.

[10] Kah M, Hofmann T (2014). Nanopesticide research: current trends and future priorities. Environ Int.

[11] Chariou PL, Dogan AB, Welsh AG, Saidel GM, Baskaran H, Steinmetz NF (2019). Soil mobility of synthetic and virus-based model nanopesticides. Nat Nanotechnol.

[12] Chariou PL, Ortega-Rivera OA, Steinmetz NF (2020). Nanocarriers for the delivery of medical, veterinary, and agricultural active ingredients. ACS Nano.

[13] Witzgall P, Kirsch P, Cork A (2010). Sex pheromones and their impact on pest management. J Chem Ecol.

[14] Yew JY, Chung H (2015). Insect pheromones: an overview of function, form, and discovery. Prog Lipid Res.

[15] Gemeno C, Yeargan KV, Haynes KF (2000). Aggressive chemical mimicry by the bolas spider *Mastophora**hutchinsoni*: identification and quantification of a major prey’s sex pheromone components in the spider’s volatile emissions. J Chem Ecol.

[16] Ghislandi PG, Beyer M, Velado P, Tuni C (2017). Silk wrapping of nuptial gifts aids cheating behaviour in male spiders. Behav Ecol.

[17] Conchou L, Lucas P, Meslin C, Proffit M, Staudt M, Renou M (2019). Insect odorscapes: from plant volatiles to natural olfactory scenes. Front Physiol.

[18] Rempel SP, Engler LG, Soares MRF, Catafesta J, Moura S, Bianchi O (2019). Nano/microfibers of EVA copolymer obtained by solution blow spinning: processing, solution properties, and pheromone release application. J Appl Polym Sci.

[19] Bhagat D, Samanta SK, Bhattacharya S (2013). Efficient management of fruit pests by pheromone nanogels. Sci Rep.

[20] Svensson GP, Lofstedt C, Skals N (2004). The odour makes the difference: male moths attracted by sex pheromones ignore the threat by predatory bats. Oikos.

[21] Zhou JJ, Litwack G (2010). Odorant-binding proteins in insects. Vitamins and hormones: pheromones.

[22] The European Commission (2019). Commission delegated regulation (EU) 2019/1702 of 1 August 2019 supplementing Regulation (EU) 2016/2031 of the European Parliament and of the Council by establishing the list of priority pests. Off J Eur Union.

[23] Marianelli L, Paoli F, Sabbatini Peverieri G, Benvenuti C, Barzanti GP, Bosio G, Venanzio D, Giacometto E, Roversi PF (2019). Long-lasting insecticide-treated nets: a new integrated pest management approach for *Popillia**japonica* (Coleoptera: Scarabaeidae). Integr Environ Assess Manag.

[24] Eilerts DF, VanderGiessen M, Bose EA, Broxton K, Vinauger C (2018). Odor-specific daily rhythms in the olfactory sensitivity and behavior of *Aedes**aegypti* mosquitoes. Insects.

[25] Wang F, Delannay C, Goindin D, Deng L, Guan S, Lu X, Fouque F, Vega-Rua A, Picimbon JF (2019). Cartography of odor chemicals in the dengue vector mosquito (*Aedes**aegypti* L., Diptera/Culicidae). Sci Rep.

[26] Andersson P, Lofstedt C, Hamback PA (2013). How insects sense olfactory patches—the spatial scaling of olfactory information. Oikos.

[27] Brito NF, Moreira MF, Melo AC (2016). A look inside odorant-binding proteins in insect chemoreception. J Insect Physiol.

[28] Wolff GH, Riffell JA (2018). Olfaction, experience and neural mechanisms underlying mosquito host preference. J Exp Biol.

[29] Raji JI, Melo N, Castillo JS, Gonzalez S, Saldana V, Stensmyr MC, DeGennaro M (2019). *Aedes**aegypti* mosquitoes detect acidic volatiles found in human odor using the IR8a pathway. Curr Biol.

[30] Nelson AS, Carvajal Acosta N, Mooney KA (2019). Plant chemical mediation of ant behavior. Curr Opin Insect Sci.

[31] Detrain C, Pereira H, Fourcassié V (2019). Differential responses to chemical cues correlate with task performance in ant foragers. Behav Ecol Sociobiol.

[32] Barbosa-Cornelio R, Cantor F, Coy-Barrera E, Rodriguez D (2019). Tools in the investigation of volatile semiochemicals on insects: from sampling to statistical analysis. Insects.

[33] Ding BJ, Hofvander P, Wang HL, Durrett TP, Stymne S, Lofstedt C (2014). A plant factory for moth pheromone production. Nat Commun.

[34] Charlton Hume HK, Vidigal J, Carrondo MJT, Middelberg APJ, Roldão A, Lua LHL (2019). Synthetic biology for bioengineering virus-like particle vaccines. Biotechnol Bioeng.

[35] Mortensen MR, Skovsgaard MB, Märcher A, Andersen VL, Palmfeldt J, Nielsen TB, Tørring T, Laursen NS, Andersen KR, Kjems J, Gothelf KV (2020). Introduction of an aldehyde handle on nanobodies by affinity-guided labeling. Bioconj Chem.

[36] Goncalves F, Ribeiro A, Silva C, Cavaco-Paulo A (2019). Release of fragrances from cotton functionalized with carbohydrate-binding module proteins. ACS Appl Mater Interfaces.

[37] Michel E, Damberger FF, Ishida Y, Fiorito F, Lee D, Leal WS, Wuthrich K (2011). Dynamic conformational equilibria in the physiological function of the *Bombyx**mori* pheromone-binding protein. J Mol Biol.

[38] Xu LQ, Neoh KG, Kang ET (2018). Natural polyphenols as versatile platforms for material engineering and surface functionalization. Prog Polym Sci.

[39] Hu K, Huang XX, Gao YQ, Huang XL, Xiao H, McClements DJ (2015). Core-shell biopolymer nanoparticle delivery systems: synthesis and characterization of curcumin fortified zein-pectin nanoparticles. Food Chem.

[40] Tang J, Sisler J, Grishkewich N, Tam KC (2017). Functionalization of cellulose nanocrystals for advanced applications. J Colloid Interface Sci.

[41] Farion IA, Burdukovskii VF, Kholkhoev BC, Timashev PS, Chailakhyan RK (2018). Functionalization of chitosan with carboxylic acids and derivatives of them: synthesis issues and prospects of practical use: a review. Express Polym Lett.

[42] De Matteis L, Alleva M, Serrano-Sevilla I, García-Embid S, Stepien G, Moros M, de la Fuente JM (2016). Controlling properties and cytotoxicity of chitosan nanocapsules by chemical grafting. Mar Drugs.

[43] Burk J, Sikk L, Burk P, Manshian BB, Soenen SJ, Scott-Fordsmand JJ, Tamm T, Tamm K (2018). Fe-Doped ZnO nanoparticle toxicity: assessment by a new generation of nanodescriptors. Nanoscale.

[44] Del Pino P, Pelaz B, Zhang Q, Maffre P, Nienhaus GU, Parak WJ (2014). Protein corona formation around nanoparticles—from the past to the future. Mater Horiz.

[45] Hartvig RA, van de Weert M, Ostergaard J, Jorgensen L, Jensen H (2011). Protein adsorption at charged surfaces: the role of electrostatic interactions and interfacial charge regulation. Langmuir.

[46] Ponnusamy L, Xu N, Nojima S, Wesson DM, Schal C, Apperson CS (2008). Identification of bacteria and bacteria-associated chemical cues that mediate oviposition site preferences by *Aedes**aegypti*. Proc Natl Acad Sci USA.

[47] Abdullah ZS, Ficken KJ, Greenfield BPJ, Butt TM (2014). Innate responses to putative ancestral hosts: is the attraction of western flower thrips to pine pollen a result of relict olfactory receptors?. J Chem Ecol.

[48] Linser PJ, Dinglasan RR, Dhadialla TS, Gill SS (2014). Insect gut structure, function, development and target of biological toxins. Insect midgut and insecticidal proteins.

[49] Caccia S, Casartelli M, Tettamanti G (2019). The amazing complexity of insect midgut cells: types, peculiarities, and functions. Cell Tissue Res.

[50] Camara MC, Monteiro RA, Carvalho LB, Oliveira JL, Fraceto LF (2020). Enzyme stimuli-responsive nanoparticles for bioinsecticides: an emerging approach for uses in crop protection. ACS Sustain Chem Eng.

[51] Song L, Wang S, Wang H, Zhang H, Cong H, Jiang X, Tien P (2015). Study on nanocomposite construction based on the multi-functional biotemplate self-assembled by the recombinant TMGMV coat protein for potential biomedical applications. J Mater Sci Mater Med.

[52] Lee KZ, Pussepitiyalage VB, Lee YH, Loesch-Fries LS, Harris MT, Hemmati S, Solomon KV (2021). Engineering tobacco mosaic virus and its virus-like-particles for synthesis of biotemplated nanomaterials. Biotechnol J.

[53] Camara MC, Campos EVR, Monteiro RA, Pereira ADS, Proenca PLD, Fraceto LF (2019). Development of stimuli-responsive nano-based pesticides: emerging opportunities for agriculture. J Nanobiotechnol.

[54] Tabatabaei PR, Hosseininaveh V, Goldansaz SH, Talebi K (2011). Biochemical characterization of digestive proteases and carbohydrases of the carob moth, *Ectomyelois**ceratoniae* (Zeller) (Lepidoptera: Pyralidae). J Asia-Pacif Entomol.

[55] Akbar SM, Sharma HC (2017). Alkaline serine proteases from *Helicoverpa**armigera*: potential candidates for industrial applications. Arch Insect Biochem Physiol.

[56] Sharifloo A, Zibaee A, Sendi JJ, Jahroumi KT (2018). Biochemical characterization a digestive trypsin in the midgut of large cabbage white butterfly, *Pieris**brassicae* L. (Lepidoptera: Pieridae). Bull Entomol Res.

[57] Souza RS, Gama MDF, Schama R, Lima JBP, Diaz-Albiter HM, Genta FA (2019). Biochemical and functional characterization of glycoside hydrolase family 16 genes in *Aedes**aegypti* larvae: identification of the major digestive beta-1,3-glucanase. Front Physiol.

[58] de Oliveira JL, Campos EVR, Germano-Costa T, Lima R, Della Vechia JF, Soares ST, de Andrade DJ, Goncalves KC, do Nascimento J, Polanczyk RA, Fraceto LF (2019). Association of zein nanoparticles with botanical compounds for effective pest control systems. Pest Manage Sci.

[59] Kaziem AE, Gao YH, Zhang Y, Qin XY, Xiao YN, Zhang YH, You H, Li JH, He S (2018). alpha-Amylase triggered-carriers based on cyclodextrin anchored hollow mesoporous silica for enhancing insecticidal activity of avermectin against *Plutella**xylostella*. J Hazard Mater.

[60] Krogh PH, Kostov K, Damgaard CF (2020). The effect of Bt crops on soil invertebrates: a systematic review and quantitative meta-analysis. Transgenic Res.

[61] Duvall LB, Ramos-Espiritu L, Barsoum KE, Glickman JF, Vosshall LB (2019). Small-molecule agonists of *Ae.**aegypti* neuropeptide Y receptor block mosquito biting. Cell.

[62] Zotti M, dos Santos EA, Cagliari D, Christiaens O, Taning CNT, Smagghe G (2018). RNA interference technology in crop protection against arthropod pests, pathogens and nematodes. Pest Manage Sci.

[63] Kim H, Shimura H, Masuta C (2019). Advancing toward commercial application of RNA silencing-based strategies to protect plants from viral diseases. J Gen Plant Pathol.

[64] Serrano-Sevilla I, Artiga Á, Mitchell SG, De Matteis L, de la Fuente JM (2019). Natural polysaccharides for siRNA delivery: nanocarriers based on chitosan, hyaluronic acid, and their derivatives. Molecules.

[65] Thach TT, Bae DH, Kim NH, Kang ES, Lee BS, Han K, Kwak M, Choi H, Nam J, Bae T (2019). Lipopeptide-based nanosome-mediated delivery of hyperaccurate CRISPR/Cas9 ribonucleoprotein for gene editing. Small.

[66] Kyrou K, Hammond AM, Galizi R, Kranjc N, Burt A, Beaghton AK, Nolan T, Crisanti A (2018). A CRISPR-Cas9 gene drive targeting doublesex causes complete population suppression in caged *Anopheles**gambiae* mosquitoes. Nat Biotechnol.

[67] Richards DM, Endres RG (2017). How cells engulf: a review of theoretical approaches to phagocytosis. Rep Prog Phys.

[68] Kacso T, Neaga IO, Erincz A, Astete CE, Sabliov CM, Oprean R, Bodoki E (2018). Perspectives in the design of zein-based polymeric delivery systems with programmed wear down for sustainable agricultural applications. Polym Degrad Stab.

[69] Production quantities of rice, paddy by country. http://www.fao.org/faostat/en/#data/QC/visualize. Accessed 1 Aug 2021.

[70] Papademetriou MK. Rice Production in the Asia-Pacific region: issues and perspectives. Bangkok: FAO; 2000. https://www.fao.org/3/x6905e/x6905e04.htm. Accessed 1 Aug 2021.

[71] Kuhnt T, Herrmann A, Benczédi D, Foster EJ, Weder C (2015). Functionalized cellulose nanocrystals as nanocarriers for sustained fragrance release. Polym Chem.

[72] Hu K, Liu S, Qiu L, Li Y (2019). Three odorant-binding proteins are involved in the behavioral response of *Sogatella**furcifera* to rice plant volatiles. PeerJ.

[73] Grillo R, Fraceto LF, Amorim MJB, Scott-Fordsmand JJ, Schoonjans R, Chaudhry Q (2021). Ecotoxicological and regulatory aspects of environmental sustainability of nanopesticides. J Hazard Mater.

[74] Elabasy A, Shoaib A, Waqas M, Shi Z, Jiang M (2020). Cellulose nanocrystals loaded with thiamethoxam: fabrication, characterization, and evaluation of insecticidal activity against *Phenacoccus**solenopsis* Tinsley (Hemiptera: Pseudococcidae). Nanomaterials.

[75] Marei N, Elwahy AHM, Salah TA, El Sherif Y, El-Samie EA (2019). Enhanced antibacterial activity of Egyptian local insects’ chitosan-based nanoparticles loaded with ciprofloxacin-HCl. Int J Biol Macromol.

[76] de Oliveira JL, Campos EVR, Pereira AES, Nunes LES, da Silva CCL, Pasquoto T, Lima R, Smaniotto G, Polanczyk RA, Fraceto LF (2018). Geraniol encapsulated in chitosan/gum arabic nanoparticles: a promising system for pest management in sustainable agriculture. J Agric Food Chem.

[77] Martel JW, Alford AR, Dickens JC (2005). Laboratory and greenhouse evaluation of a synthetic host volatile attractant for Colorado potato beetle, *Leptinotarsa**decemlineata* (Say). Agric For Entomol.

[78] Wen G, Khelifi M, Cambouris AN, Ziadi N (2018). Responses of the Colorado potato beetle (Coleoptera: Chrysomelidae) to the chemical composition of potato plant foliage. Potato Res.

[79] Faraldos JA, Coates RM, Giner JL (2013). Alternative synthesis of the colorado potato beetle pheromone. J Org Chem.

[80] Balasko MK, Mikac KM, Bazok R, Lemic D (2020). Modern techniques in Colorado potato beetle (*Leptinotarsa**decemlineata* Say) control and resistance management: history review and future perspectives. Insects.

[81] Strandberg MT, Scott-Fordsmand JJ (2002). Field effects of simazine at lower trophic levels—a review. Sci Total Environ.

[82] Strandberg M, Scott-Fordsmand JJ (2004). Effects of pendimethalin at lower trophic levels—a review. Ecotoxicol Environ Saf.

[83] Amorim MJB, Rombke J, Scheffczyk A, Soares A (2005). Effect of different soil types on the enchytraeids *Enchytraeus**albidus* and *Enchytraeus**luxuriosus* using the herbicide Phenmedipham. Chemosphere.

[84] Frampton GK, Jansch S, Scott-Fordsmand JJ, Rombke J, Van den Brink PJ (2006). Effects of pesticides on soil invertebrates in laboratory studies: a review and analysis using species sensitivity distributions. Environ Toxicol Chem.

[85] Novais SC, Soares A, Amorim MJB (2010). Can avoidance in *Enchytraeus**albidus* be used as a screening parameter for pesticides testing?. Chemosphere.

[86] Goulson D, Nicholls E, Botias C, Rotheray EL (2015). Bee declines driven by combined stress from parasites, pesticides, and lack of flowers. Science.

[87] Paliwal R, Palakurthi S (2014). Zein in controlled drug delivery and tissue engineering. J Control Release.

[88] Oehlke K, Keppler JK, Milsmann J, Mayer-Miebach E, Greiner R, Steffen-Heins A (2019). Adsorption of beta-lactoglobulin to solid lipid nanoparticles (SLN) depends on encapsulated compounds. J Food Eng.

[89] Thrane S, Janitzek CM, Matondo S, Resende M, Gustavsson T, de Jongh WA, Clemmensen S, Roeffen W, van de Vegte-Bolmer M, van Gemert GJ (2016). Bacterial superglue enables easy development of efficient virus-like particle based vaccines. J Nanobiotechnol.

